# Metal nanoparticles as a promising technology in targeted cancer treatment

**DOI:** 10.1080/10717544.2022.2039804

**Published:** 2022-02-25

**Authors:** Jia-Jie Xu, Wan-Chen Zhang, Ya-Wen Guo, Xiao-Yi Chen, You-Ni Zhang

**Affiliations:** a Department of Head and Neck Surgery, Otolaryngology & Head and Neck Center, Cancer Center, Zhejiang Provincial People’s Hospital (Affiliated People’s Hospital, Hangzhou Medical College), Hangzhou, China; b Key Laboratory of Endocrine Gland Diseases of Zhejiang Province, Hangzhou, China; c Second Clinical Medical College, Zhejiang Chinese Medical University, Hangzhou, China; d Clinical Research Institute, Zhejiang Provincial People’s Hospital, Affiliated People’s Hospital of Hangzhou Medical College, Hangzhou, China; e Department of Laboratory Medicine, Tiantai People’s Hospital of Zhejiang Province (Tiantai Branch of Zhejiang People’s Hospital), Taizhou, China

**Keywords:** Cancer treatment, metal nanoparticles, targeting, radiation therapy, hyperthermia

## Abstract

Traditional anticancer treatments have several limitations, but cancer is still one of the deadliest diseases. As a result, new anticancer drugs are required for the treatment of cancer. The use of metal nanoparticles (NPs) as alternative chemotherapeutic drugs is on the rise in cancer research. Metal NPs have the potential for use in a wide range of applications. Natural or surface-induced anticancer effects can be found in metals. The focus of this review is on the therapeutic potential of metal-based NPs. The potential of various types of metal NPs for tumor targeting will be discussed for cancer treatment. The *in vivo* application of metal NPs for solid tumors will be reviewed. Risk factors involved in the clinical application of metal NPs will also be summarized.

## 1. Introduction

**Figure 1.**
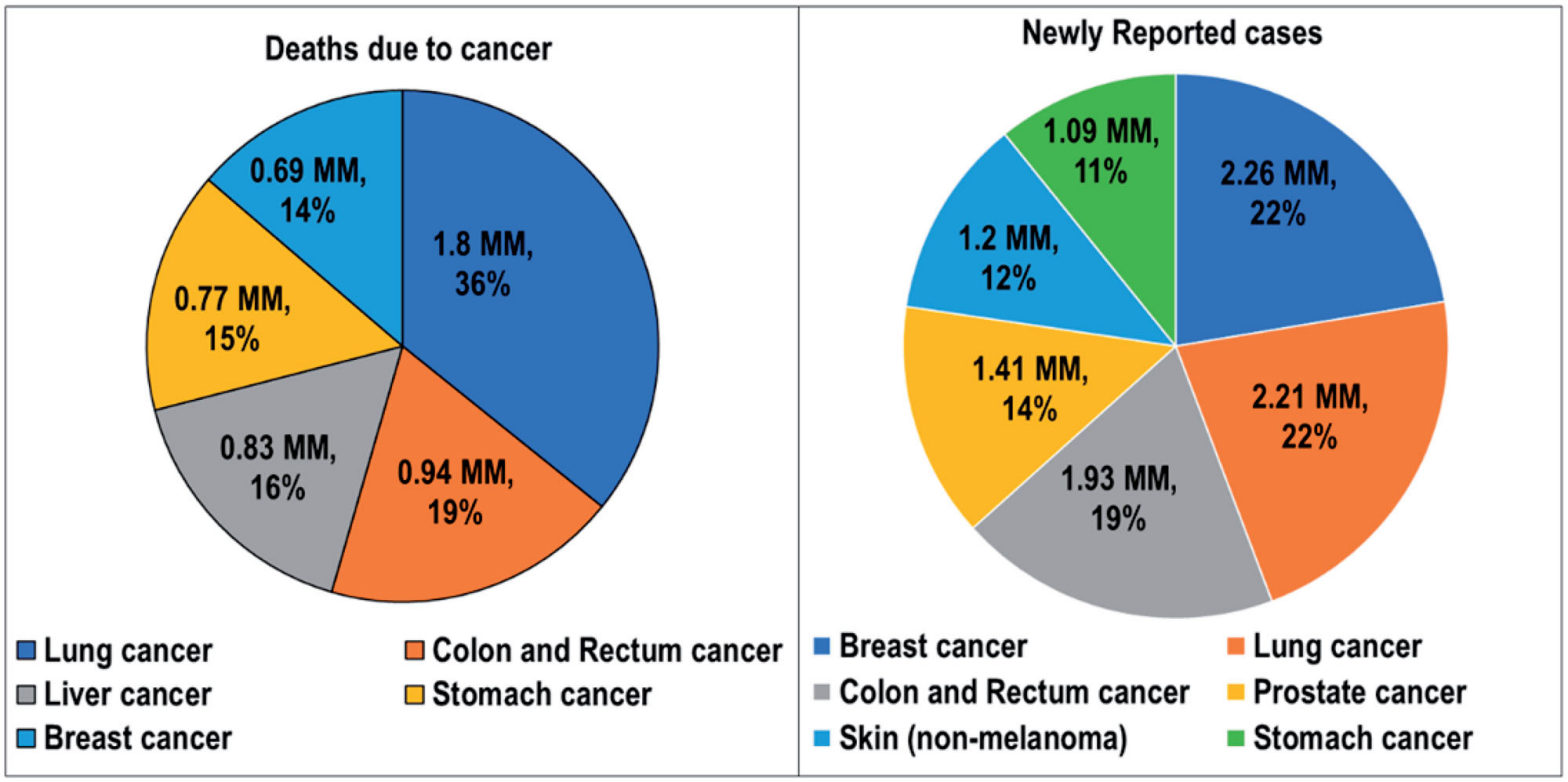
Statistics on the deaths due to cancer and newly reported cancer cases in the year 2020. MM: millions of people.

Cancer is a condition in which abnormalities in the genome arise. These changes trigger unregulated cell division, resulting in tissue damage (Pugazhendhi et al., 2018). Cancer is a multifactorial disease because of its complex combination of hereditary and environmental components. DNA damage is the primary cause of the abnormalities responsible for cancer (Rivas-Domínguez et al., 2021). Cancer has the highest clinical, social, and economic burden of any human disease in terms of cause-specific disability-adjusted life years (DALYs). The overall risk of developing cancer for people aged 0–74 years is 20.2% (22.4% in men and 18.2% in women, respectively) (Mattiuzzi & Lippi, 2019). Sung et al. reported alarming global cancer statistics for the year 2020. Globally, an estimated 19.3 million new cancer cases (18.1 million excluding nonmelanoma skin cancer) and almost 10.0 million cancer deaths (9.9 million excluding nonmelanoma skin cancer) occurred in 2020. As the most diagnosed cancer, female breast cancer exceeded lung cancer. Breast cancer had 2.3 million new cases (11.7%), followed by lung (11.4%), colorectal (10.0%), prostate (7.3%), and stomach (5.6%) cancers. Lung cancer remained the leading cause of cancer death, with an estimated 1.8 million deaths (18%), followed by colorectal (9.4%), liver (8.3%), stomach (7.7%), and female breast (6.9%) cancers (Sung et al., 2021). According to a report by the World Health Organization (WHO), cancer was the primary cause of mortality worldwide, contributing to about 10 million deaths in 2020. A statistical overview of newly reported cancer cases and deaths reported are presented in Figure 1 (Cancer, 2022).

The most prevalent cancer therapies are chemotherapy, radiation, and surgery (Singhal et al., 2010). One of the most frequent cancer treatments is chemotherapy. Conventional chemotherapy mainly kills cancer cells by blocking mitosis and preventing DNA synthesis. Unintended and sometimes fatal side effects may occur when chemotherapeutic drugs target healthy tissues, particularly those that are rapidly expanding like blood and digestive tract cell membranes (Hossen et al., 2019). Traditional chemotherapy must be supplemented or replaced with less harmful systems due to the harm that conventional chemotherapeutic agents cause to healthy cells (Yan et al., 2020). Similarly, radiotherapy has many disadvantages as the radiations are also delivered to the healthy tissues surrounding the tumor and have the potential to harm them (Çeşmeli & Avci, 2019). Surgical therapy is not only costly but also a very complicated procedure. It requires specific expertise as well. Surgical therapy may also necessitate additional clinic visits due to suture removal, infection, contact dermatitis, spitting sutures, and graft check, among other things. Furthermore, many elderly patients require additional care after surgery, such as dressing changes, transportation, and follow-up visits, which adds to the cost of surgical therapy (Ahluwalia et al., 2020).

**Figure 2.**
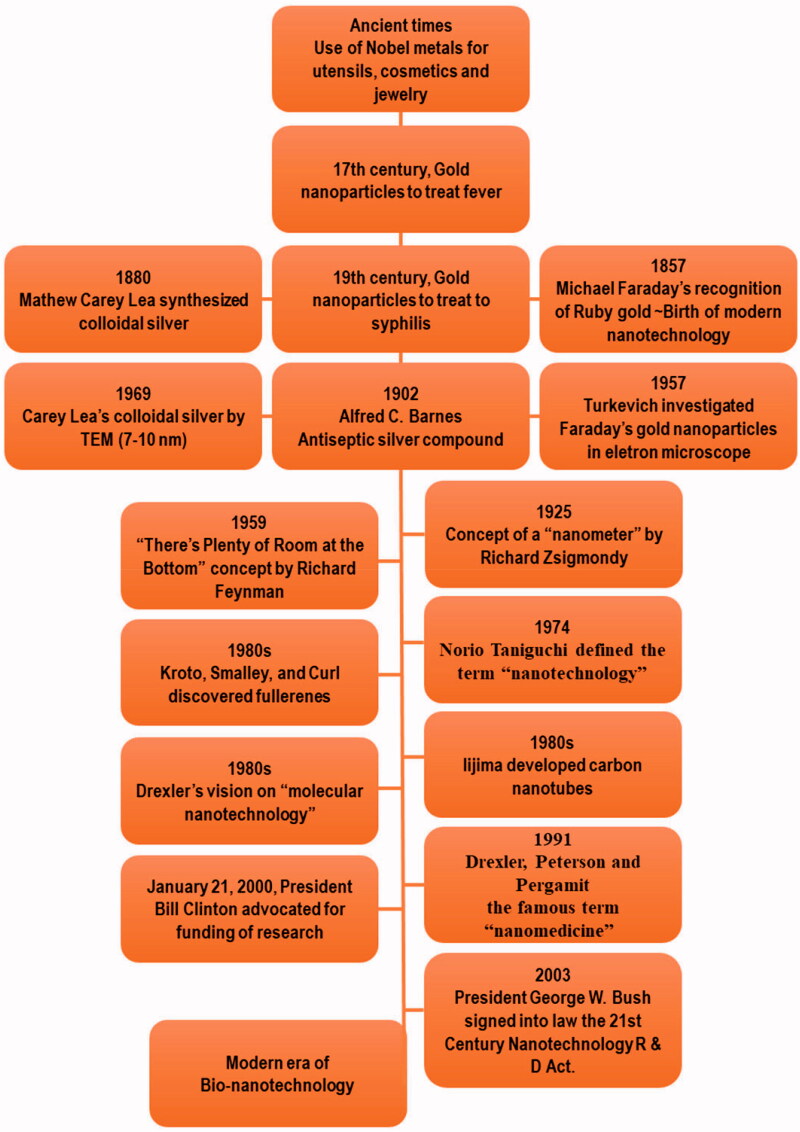
Timeline of the advancement in nanotechnology.

**Figure 3.**
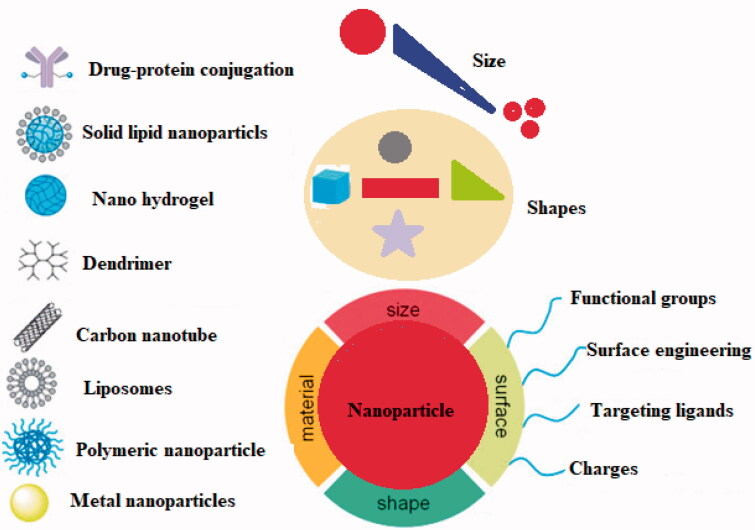
NPs categorized based on their size, material, surface, and shape.

Nanotechnology has opened a new era in every field of life. The timeline of the advancement in nanotechnology has been represented in Figure 2. The ability of nanotechnology to detect a wide range of molecular signals and biomarkers in real-time is just what drives breakthroughs in early detection, diagnostics, prognostics, and therapeutic strategy. Cancer nanotherapeutics have overcome several shortcomings of conventional therapies, including nonspecific biodistribution, low water solubility, and poor bioavailability. It provides high sensitivity, specificity, and multiplexed measurement capacity (Zhang et al., 2019). A nanoparticle (NP) is a particle that has dimensions between 1 and 100 nm. When compared to the traditional drug delivery systems, NP-based drug delivery systems have higher efficacy due to increased half-life of vulnerable drugs and proteins, improved solubility of hydrophobic drugs, and the ability to control and target drug release in diseased sites (Dang & Guan, 2020). The types and characteristics of NPs based on size (1–100 nm), type (metal or polymeric), shape, and targeting are depicted in Figure 3.

**Figure 4.**
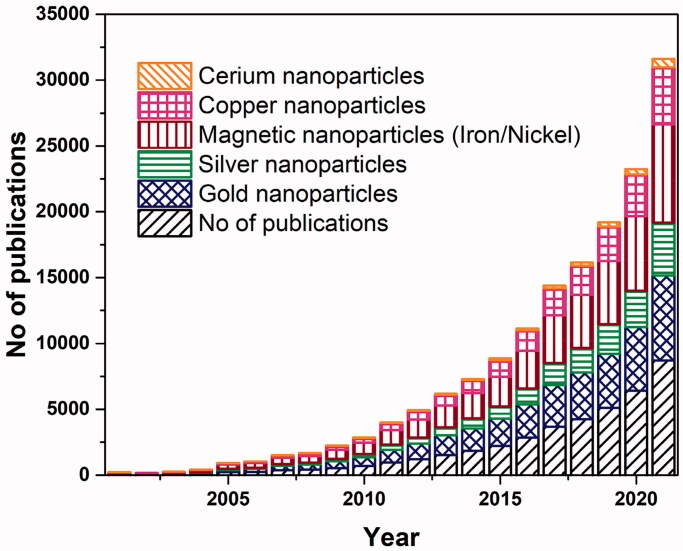
Number of publications for the year 2001–2021. Data retrieved from sciencedirect.com with search terms ‘metal’, or respective ‘metal name’, ‘nanoparticle’, and ‘cancer’ on January 22 2022.

Metal NPs have gained specific attention among all the NPs because these have the potential to serve as multipurpose agents. Gold, silver, iron and/or iron oxide, zinc, titanium, cerium oxide, nickel, copper, magnesium, barium, calcium, and bismuth-based metal NPs have been reported as a cancer treatment. The leading role of metal NPs in the current research platforms against cancer, and the research interest in this topic is increasing day by day, as can be depicted in Figure 4. The comparison of five commonly synthesized metal NPs showed that gold NPs are among the leading, followed by silver and magnetic nanoparticles (MNPs). Several studies have shown metal NPs can be used to treat cancer and preliminary and clinical trials are now underway. The non-noble metal-based cancer therapy can progress toward cost-effective treatment as compared to expensive chemotherapy.

In this review, the role of metal NPs in cancer therapy will be discussed. It will be examined how metal NPs reach the target areas by exploiting either the leaky vasculature within the tumor or targeting the overexpressed receptors on the tumor cells. The purpose of this article is to enlighten researchers, particularly those working on nanotechnology-based cancer therapy, about the potential of metal NPs in cancer treatment. The advantages of noble and non-noble metals and their drawbacks will be highlighted. The clinical potential of metal NPs will be determined by their efficacy against cancer cell lines and *in vivo* tumors. The last part will deliberate the toxicity concerns of the metal NPs in clinical application.

## 2. Mechanism of cancer-targeting

**Figure 5.**
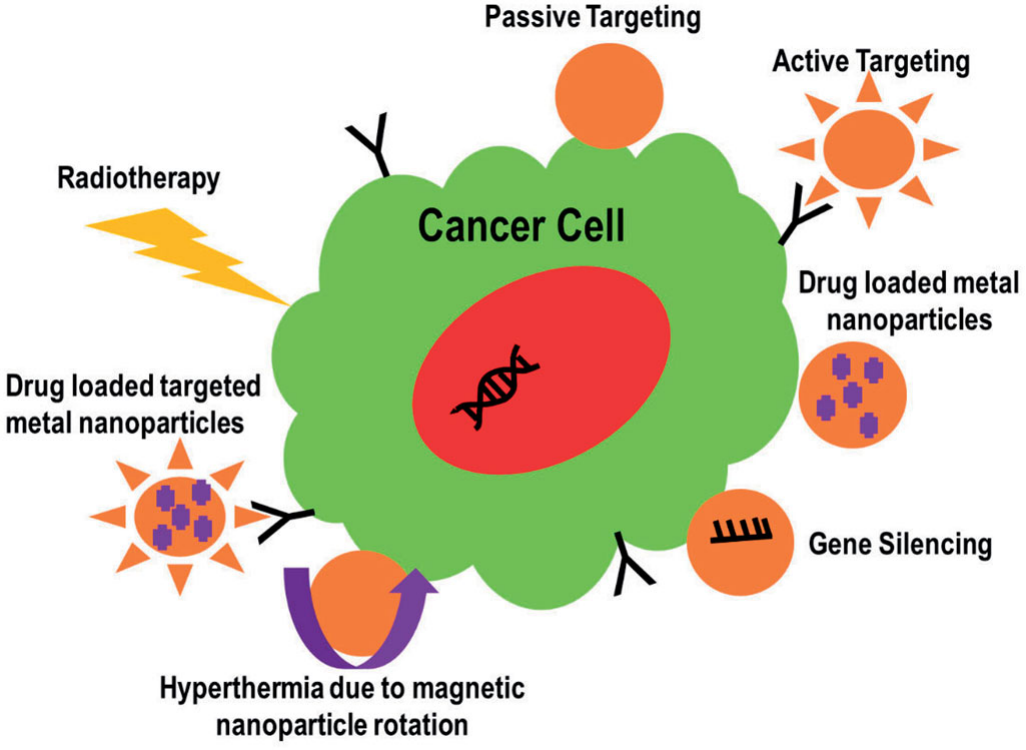
Mechanisms involved in cancer targeting.

Metal NPs have been reported to have antitumor properties. The mechanisms of action involved in cancer therapeutics have been discussed below. Figure 5 illustrates these mechanisms.

### 2.1. Active or passive targeting of tumor

Therapeutic agent accumulation can be enhanced by metal NP treatments in two ways: passive and active. For cancer treatment, abnormal branching and leaky sites with pore sizes ranging from 100 nm to several hundred nanometers are commonly found in tumor vasculatures. The reason is the rapid development of endothelial cells reduced the number of pericytes (Lok et al., 2014). Due to this leaky vasculature, the body concentrates inert metal NPs in the tumor under the passive targeting mechanism. This is referred to as the increased permeability and retention (EPR) effect. On the other hand, active targeting enhances the therapeutic delivery system by functionally modifying the metal NP's surface, resulting in selective tissue targeting (Pissuwan et al., 2006). The surface modification of NPs with tumor-targeting ligands such as antibodies, folic acid, and peptides, or the incorporation of tumor-targeting ligands into NPs via disulfide bonding, might result in targeted intra-tumor drug release (Wang et al., 2012). In a study, silver NPs functionalized with a fluorescent cyclic arginine–glycine–aspartic acid (RGD) peptide were found to be effective in identifying and targeting cancerous cells. Researchers found that the RGD peptide sequence can interact specifically with integrins, which are critical receptors for cancer growth and bacterial adhesion/invasion. Immobilization of a fluorescent RGD peptide onto silver NPs, with a diameter of 13 nm, was used to functionalize the hybrid peptide–metal NPs biointerface. Cellular uptake by SH-SY5Y neuroblastoma and K562 chronic myelogenous leukemia cells was confirmed by confocal microscope imaging (Di Pietro et al., 2016).

### 2.2. Tumor targeting through gene silencing

The regulation of gene expression in a cell to prohibit the expression of a specific gene is known as gene silencing. Due to its potential to suppress genes implicated in tumor formation, gene silencing grasps promising cancer therapy gene silencing is the process of altering gene expression on an epigenetic level. This is accomplished mostly through the use of antisense DNA and short interfering RNA (Liu et al., 2019). Fernandes & Baptista (2017) reported gene silencing using multifunctional gold NPs for cancer therapy to improve tumor cell identification and uptake. Gold NPs' surfaces were functionalized using targeting peptides. This approach inhibits KRAS gene expression in colorectal cancer cell lines while leaving healthy fibroblasts unharmed. Gene silencing with small interfering RNA (siRNA) is another option. siRNAs can be delivered to cells by using a platelet cell membrane-coated metal (zinc)-organic framework (MOF). Using a simple one-pot method, synthetic siRNAs were loaded onto porous metal-organic framework (MOF) NPs. pH affects the structural integrity of MOF scaffolds. *In vitro* targeting and intracellular localization were performed on human SK-BR-3 breast cancer cells (HTB-30; American Type Culture Collection, Manassas, VA). To bind specifically to cancer cells, a platelet membrane coating was employed (Fernandes & Baptista, 2017).

### 2.3. Drug delivery through nanoparticles

Cancer drugs in clinical practice are low molecular weight chemicals that can diffuse easily into healthy tissues as well as tumor tissues. This results in an even distribution throughout the body, a short half-life, and a rapid clearance rate. The amount of medicine that reaches the target site is very low, lowers therapeutic efficacy, and increases the risk of side effects, including the possibility of drug resistance (Skoetz et al., 2017; Farzin et al., 2020). Metal NPs can act as vectors for targeted drug delivery. Both hydrophobic drugs, e.g. paclitaxel loaded selenium NPs (Bidkar et al., 2017) and hydrophilic drugs, e.g. doxorubicin (DOX) loaded iron oxide (Fe_3_O_4_@SiO_2_@mSiO_2_) NP drug delivery system (Gao et al., 2018) can be loaded into metal NPs. The small size and stability of the metal NPs enhance the bioavailability of the drugs. Metal NP formulation containing a superparamagnetic iron oxide core coated with short- and long-chain polyethylene glycol (PEG) was reported. The hydrophobic paclitaxel and folic acid were coupled to a metallic core, and the PEG served as a hydrophilic outer layer. When compared to free paclitaxel, the NPs system delivered the drug at conditions that mimicked the acidic intracellular pH of breast cancer cells, and the folic conjugation resulted in higher NP absorption by target cells, which increased the cytotoxicity to target cells (Jeon et al., 2019). In another study, for colon target dual drug delivery, a chitosan/palladium nanocomposite was created. Curcumin (CUR) and 5-fluorouracil (5-FU) were loaded individually and in combination with chitosan/Pd nanocomposite. The growth of HT-29 cells is inhibited more effectively by co-encapsulated nanocomposite than by 5-FU, CUR monotherapy (Dhanavel et al., 2017). Some NP systems have higher IC_50_ values as compared to the free drug because of slow drug release, but the systems show sufficient credibility as targeted drug delivery systems (Pan et al., 2017). The comparison of IC_50_ values of the anticancer drugs-based metal NPs and their comparison with the free drug is given in Table 1.

**Table 1.** Comparison of IC_50_ values of the anticancer drugs-based metal nanoparticles and their comparison with the free drug.

Nanoparticle type	Cancer cell line	IC_50_ value of nanoparticle system	Anticancer drug	IC_50_ value of free drug	References
Resveratrol stabilized gold nanoparticles	Glioma carcinoma cell line (LN 229)	4 μg/mL	Doxorubicin	6 μg/mL	Mohanty et al. (2014)
Multifunctional nanosystem (MFNCs) of gold nanorods, iron oxide nanoparticles, and gold nanoclusters assemblies within BSA nanoparticles	HeLa cancer cells	2.3 μg/mL	Doxorubicin	0.5 μg/mL	Pan et al. (2017)
Gold nanoparticles based nanoconjugates	Lung cancer cell line (H520)	25 µM	Docetaxel	38 µM	Thambiraj et al. (2019)
Gold nanoparticles	Breast cancer cells MCF-7	30 ± 5 μg/mL	Chloroquine	>30 ± 5 μg/mL	Joshi et al. (2012)
Silver nanoparticle	HepG2 cells	1.92 μg/mL	Epirubicin	0.11 μg/mL	Ding et al. (2019)
Jacalin-capped silver nanoparticles	Human chronic myeloid leukemia	100 nM	Acetylshikonin (AS) and beta-dimethylacrylshikonin (BDS)	500 nM	Ayaz Ahmed et al. (2016)
PEG capped silver nanoparticles	Breast cancer cells MCF-7	258.6 μg/mL	Methotrexate	512.7 μg/mL	Muhammad et al. (2016)
Copper nanoparticles based systems	Drug-resistant prostate cancer cell	85, 172, and 193 nM	Paclitaxel (control)	2575 nM	Chen et al. (2018)

### 2.4. Nanoparticles based hyperthermia

When biological materials are heated just a few degrees beyond their normal temperature, significant changes occur, including ceil death. Hyperthermia therapy is a type of cancer treatment in which bodily tissue is subjected to high temperatures, usually up to 113 degrees Fahrenheit (40–45 °C), in order to harm or kill cancer cells (Jose et al., 2020). All hyperthermia-related events aim to change the extracellular milieu by triggering immune responses and driving tumor cells to switch to an anaerobic metabolic system (Balivada et al., 2010). Hyperthermia is divided into three categories based on the site of application: whole-body hyperthermia, regional hyperthermia, and localized hyperthermia. Depending on the form of application, whole-body hyperthermia can be invasive or noninvasive. *Invasive hyperthermia* is the process of heating blood extracorporeally, whereas noninvasive hyperthermia is the process of increasing temperature through the use of hot air, hot wax, or RF or IR irradiation, which cannot be used to treat deep tumors (Wust et al., 2002). Hyperthermia in a specific area called *regional hyperthermia* uses noninvasive treatment (e.g. non-ionizing electromagnetic radiation (NIR) or ultrasound are used to heat regionally situated tumors) or invasive approaches (e.g. thermal conduction, or the use of magnetic implants) (Baker et al., 1982; Longo et al., 2016). The invasive or noninvasive procedures listed above are utilized to heat tiny tumors to a depth of 4 cm in the *localized hyperthermia* approach (Jose et al., 2020). Nanotechnology especially MNPs has created a huge opportunity to advance the field of hyperthermia. The heat dissipation processes of MNPs can be used to understand the mechanism of tumor-killing by hyperthermia. Magnetic NPs disperse heat to tumor cells through two processes: Neel relaxation and Brownian relaxation. Neel relaxation occurs when the magnetic moment reorients parallel to the applied magnetic field, whereas Brownian relaxation happens when the nanomaterial is mechanically rotated toward the external magnetic field. When a material is exposed to an external alternative magnetic field (AMF) with a magnetic field reversal time less than the material's magnetic relaxation period, both processes occur (Chen et al., 2017). High-grade superparamagnetic MnFe_2_O_4_ NPs were manufactured using a low-cost and environmentally friendly co-precipitation approach with the goal of hyperthermia application. The results showed that MnFe_2_O_4_ MNPs could reach hyperthermia temperature (42 °C) in 260 seconds at a low level of 0.4 g/mL, indicating that the material could be employed as a heating agent in magnetic hyperthermic treatment (Patade et al., 2020). Ma et al. reported Fe_3_O_4_–Pd Janus NPs with amplified dual-mode hyperthermia and enhanced ROS generation for breast cancer treatment. Under alternating magnetic field (AMF) plus laser irradiation, Fe_3_O_4_–Pd JNPs achieved a larger temperature enhancement than the corresponding individual modality (only AMF or laser irradiation alone for Fe_3_O_4_–Pd JNPs) or the total of two individual modalities. In the presence of H_2_O_2_ in an acidic environment, Fe_3_O_4_–Pd JNPs increased ROS generation due to the interface synergistic effect in creating hydroxyl radicals (OH), which was realized by Fe_3_O_4_ NP-based Fenton reaction and Pd nanosheet-based catalytic capabilities. Surprisingly, with external AMF + laser irradiation, the ROS level was raised even higher. On an orthotopic mouse breast cancer model, the anti-tumor activity of Fe_3_O_4_–Pd JNPs was tested *in vivo*. Under AMF with laser irradiation, guided by MRI/PA dual-mode imaging with excellent spatial resolution and precision, Fe_3_O_4_–Pd JNPs provided full tumor suppression without notable deleterious effects (Ma et al., 2019).

### 2.5. Radiotherapy treatment through nanoparticles

High-energy radiations are used in radiation therapy (RT) to inhibit the proliferation or kill malignant cells. Ionizing radiations are the type of primary concern for cancer treatment. Ionizing radiation is electromagnetic radiation with sufficient energy to ionize or remove electrons from atoms or molecules, resulting in the formation of ions. Ions with high kinetic energy collide several times, depositing a considerable amount of energy in the cells they travel through. The transferred energy is sufficient to stop tumor cells from replicating DNA or transcribing RNA, resulting in cell death. The most difficult aspect of radiotherapy is delivering a deadly dosage of radiation to tumor cells while preventing unintended cell damage (Fard et al., 2017; Song et al. 2017; Césaire et al. 2018; Richardson et al., 2018; Carozza et al. 2020). Metal NPs are widely employed in radiotherapy to increase the specificity of radiations to the targeted spot, reducing radiation dose and preventing toxicity and injury to normal tissues. Ionizing radiation causes the radiolysis of water molecules, resulting in the production of reactive oxygen species (ROS). They have a significant damaging effect on DNA due to the unpaired electron. Metal NPs use a variety of ways to improve radiation targeting. Metals increase tumor cell oxidative stress, promote selective apoptosis, and reduce clonogenic survival (Al-Musywel & Laref 2017; Choi et al., 2020; Igaz et al., 2020; Schuemann et al., 2020). Various metal NPs have been used in recent RT research; however, silver and gold NPs surpass other metal NPs for radio sensitization applications in cancer imaging and therapy due to their high atomic number and mass-energy coefficient. To cope with cancer, a synergistic treatment of ionization and hyperthermia is also effective. Simultaneous treatment that causes hyperthermia in tumor cells while also delivering radiations appears perfect, but hyperthermia prior to radiations is thought to be more successful. Inverse Metal NPs improve radiation targeting while also causing a hyperthermic response at the tumor location. When heat is combined with metal and RT for cancer, response rates increase by 16–26% (Cędrowska et al., 2020; Tolkaeva et al., 2021).

Sears et al. have shown that triple-negative breast cancer is sensitive to photothermal and ionizing radiation. Nanoparticles of silver having a peak absorbance in the near-infrared (NIR) spectrum were produced. The scientists tested the possibility of treating MDA-MB-231 TNBC cells selectively without harming nonmalignant MCF-10A breast cells using a multimodal method based on combination photothermal therapy, IR sensitization, and targeted cytotoxicity. There were no nonmalignant mammary epithelial cells saved as a result of this combination. It was shown that thermal radiation sensitization using triangular silver NPs resulted in excellent results despite the lower treatment dose and frequency (Sears et al., 2021), excellent results despite the lower treatment dose and frequency (Sears et al., 2021). When combined with the histone deacetylase inhibitor SAHA, gold NPs and radio sensitization were tested in 2D and 3D cancer cell cultures. Radiation-resistant A549 and DU-145 cancer cells were used to test the treatment's effectiveness. Prior to radiotherapy, gold NPs and SAHA dramatically reduced the number of live cells, which indicates that the combination of gold NPs and SAHA significantly boosted the potency of irradiation (Igaz et al., 2020).

## 3. Metal nanoparticles used for cancer therapy

Various metal NPs have been employed in cancer treatment. The metal NPs can be divided into noble and non-noble metals-based NPs. The role of some of the metal NPs in cancer treatment is highlighted in Table 2.

**Table 2.** Potential of metal nanoparticles as cancer treatment.

Type of metal nanoparticles	Targeted cancer	Targeting role	References
Glyco-gold nanoparticles	Cervical cancer (HeLa and L929 cells)	Conjugated RNase	Zhao et al. (2021)
Silver nanoparticles	Pancreatic cancer (PANC-1, AsPC-1, and MIA PaCa-2 cells)	Oxygen based (ROS) or nitrogen based (RNS) reactive species	Wang et al. (2021)
Mesoporous platinum nanoparticles	Breast cancer (MCF-7/ADR cells)	Photothermal therapy/doxorubicin	Fu et al. (2020)
Superparamagnetic iron oxide nanoparticles	Breast cancer bone metastasis (MDA-MB-231/ bone marrow cells)	Bone-targeting peptide-conjugated	Pang et al. (2021)
Nickel oxide nanoparticles	Triple-negative breast cancer cells (Vero cell line and MDA-MB-231 cells)	Folic acid decorated and pH-sensitive	Binu et al. (2021)

### 3.1. Noble metals-based nanoparticles for cancer therapeutics

The noble metal is the metal from any of the several metallic chemical elements that have outstanding oxidation resistance, even at high temperatures. The main limitation with their application is the high cost of these metals. Noble metals commonly employed for NPs preparation include gold, silver, platinum, and palladium.

#### 3.1.1. Gold nanoparticles

**Figure 6.**
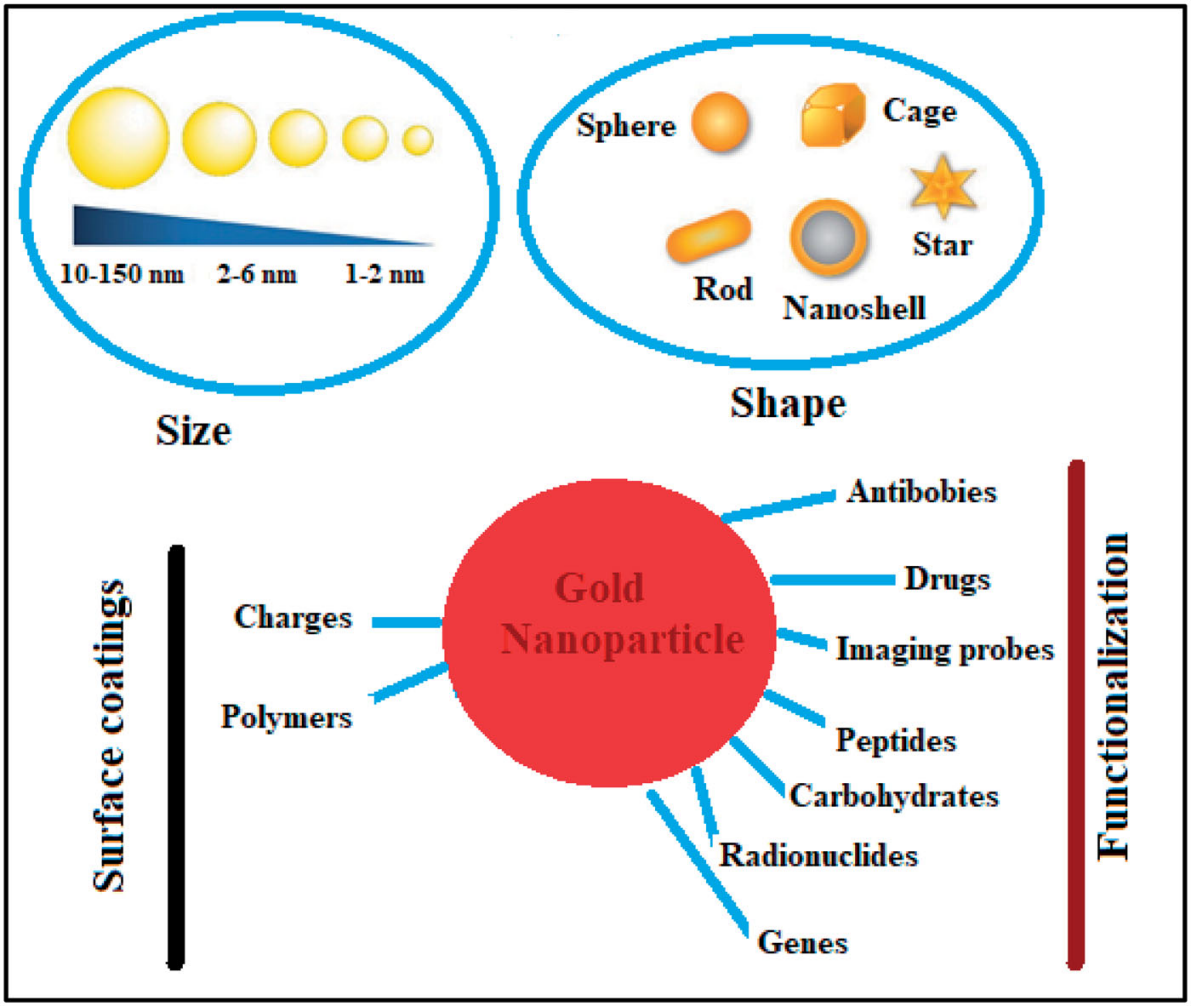
Gold nanoparticles physicochemical properties and functionalization against cancer.

The non-reactive nature of gold makes it a noble element. Its resistance to chemical oxidation renders it impervious to degradation and corrosion. Thus, it can retain its form and luster for millennia. Gold NPs can be made in a variety of ways, including chemical, physical, biological, and green synthesis. The bottom-up and top-down approaches are used in all types. Gold NPs have a wide variety of biomedical applications due to their unique physicochemical characteristics (Chen et al., 2013). Recently, there has been a lot of discussion about tumor targeting. Gold-based metal NPs system along with their cancer-targeting properties have been elaborated in Figure 6. The anti-tumor effects of gold NPs can be further enhanced by surface functionalization or coating, and these NPs can be used for a variety of diagnostic, therapeutic, bioimaging, and prognostic purposes.

Botteon et al. reported the biosynthesis of gold NPs and used a bee product called Brazilian red propolis (BRP). T24 bladder cancer and PC-3 prostate cancer cell lines were treated with biosynthetic gold NPs and exhibited substantial *in vitro* cytotoxic effects (Bray et al., 2018). Curcumin and isonicotinic acid hydrazide corona functionalized gold NPs were formulated to target cancer, according to Umapathi et al. (2020). Lung cancer cells (LK-2) and fibrillary epithelial cells (TIG-120) are particularly sensitive to the harmful effects of functional NPs. The generation of ROS by conjugating CUR and INH on AuNPs increased anticancer activity (ROS). When the investigation was extended, apoptosis and morphological changes in LK-2 and TIG-120 cells were identified. Additionally, the anticancer efficacy of these NPs is contrasted with that of traditional cisplatin (Botteon et al., 2021).

#### 3.1.2. Silver nanoparticles

Silver NPs are becoming increasingly popular in biomedicine due to the wide range of applications they have, such as antimicrobial wound dressings, topical lotions to prevent infection, and anticancer therapies (Sondi & Salopek-Sondi, 2004). The primary mechanisms through which silver NPs function include ROS, oxidative stress, and DNA damage. ROS are essential for the survival of cells since they help to keep their internal balance in check. As a byproduct of cellular metabolism, ROS is a key player in signaling networks within cells. However, an excessive amount of intracellular ROS damages DNA, lipids, and proteins as a mechanism for silver NP-induced toxicity (Jain et al., 2021). Toxicity in treated cells is caused by the release of silver ions in the cytosol following endocytosis of silver NPs and their breakdown in an acidic environment. Thus, silver NPs have been linked to an increased risk of cancer and cell death due to their ability to interfere with the cell's basic metabolic and cell cycle pathways (De Matteis et al., 2015). Silver NPs functionalized paclitaxel nanocrystals boost the overall anti-cancer activity on human cancer cells, according to Muhammad et al. (2021). Nanocrystals were developed that combined the organic anti-cancer drug paclitaxel with inorganic silver NPs as tumor-targeting agents. The polydopamine (PDA) was applied to the paclitaxel nanocrystals, which were used as a template. Silver NPs and the tumor-targeting peptide NR1 were grafted onto the PDA layer, which was also a connecting bridge for manufacturing and depositing the silver NPs *in situ* (RGDARF). Drug nanocrystals coated with NR1/AgNP demonstrated dramatically improved cellular absorption efficiency, *in vitro* anti-cancer activity and an anti-migration effect against a range of cancer cells as a result. According to the data, silver NPs and paclitaxel had an additive or synergistic influence on each other, as well as on the NR1-receptor interaction, pH-responsive drug release, and the small size. In terms of selectivity and biocompatibility, these NR1-AgNP-decorated PTX nanocrystals were very well-balanced. The apoptotic efficacy of these nanocrystals was also high, resulting in lysis of the cell membrane as well as damage to the nucleus, dysfunction of the mitochondria, excessive production of ROS, and double-stranded DNA breakage. Authors proposed that P53 and caspase 3 activation, as well as Bax-to-Bcl-2 ratio modification, may be relevant to the putative acting mechanism and molecular basis of these distinct pharmacological nanocrystals (Umapathi et al., 2020; Muhammad et al., 2021).

#### 3.1.3. Platinum nanoparticles

Patients are treated all around the world using platinum-based drugs like cisplatin, carboplatin, and oxaliplatin. However, the lack of specificity in cancer treatment leads to adverse effects and an increase in drug resistance (Mochida et al., 2017). Biotechnology, nanomedicine, and pharmacology all use platinum NPs in respective studies. Inorganic platinum NP nanoformulations are yet to be tested in humans. The longer the platinum NPs can circulate inside the body, the more beneficial it may be to coat their surfaces with a biocompatible substance like polyvinylpyrrolidone (PVP) (Jeyaraj et al., 2019). In a study, DOX was used as a model drug, to make PVP-functionalized platinum NPs with an octopod shape that demonstrated mono-dispersity. The system was used to improve drug distribution and reduce toxicity. It was found that both drug release and biocompatibility were improved with the platinum–DOX conjugate system. Two intrinsic breast cancer cell subtypes (MCF-7 and MDA-MB-231) were used to assess the system's cytotoxic capacity. The mechanisms involved in inhibiting the PI3K/AKT signaling pathway via activation of the tumor suppressor gene PTEN were found to be implicated (Patel et al., 2021). Kankala et al. postulated that platinum NPs can penetrate deep into tumors and have synergistic therapeutic effects because of the free radical species-assisted catalysis of platinum NPs. The ultrasmall platinum NPs were disseminated in chitosan loaded across zinc-doped mesoporous silica nanocarriers through a self-assembly mechanism. Doxorubicin molecules were loaded more efficiently into the tumor's acidic microenvironment by the zinc species doped in the siliceous frameworks, without the need for any additional functionalization. This strategy improved anticancer efficacy by dismantling the established coordination interactions between the host and guest species (Dhavale et al., 2021). The technique considerably aided tumor eradication by facilitating deep tumor penetration and simultaneously generating detrimental free radicals for the destruction of MDR malignancies (Kankala et al., 2020).

#### 3.1.4. Palladium nanoparticles

**Figure 7.**
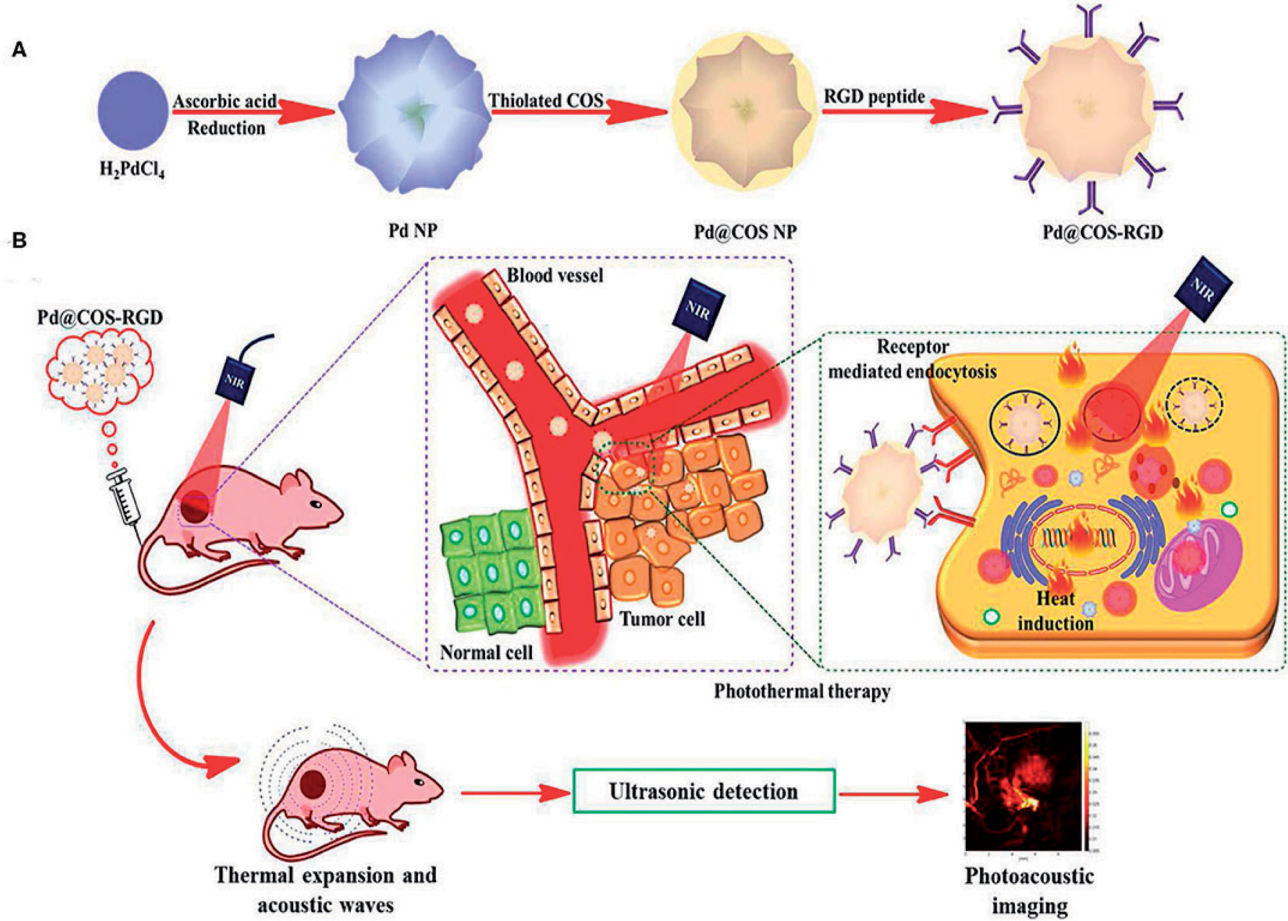
Preparation (A), functionalization and tumor targeting (B) of palladium nanoparticles (Bharathiraja et al., 2017).

Palladium NPs can be used in theragnostic applications because of their outstanding catalytic and optical capabilities. Palladium nanomaterial has been used as a prodrug activator, photothermal agent, and anticancer and/or antibacterial therapy, according to researchers. The multifunctional palladium NPs mediating photothermal therapy along with imaging have been presented in Figure 7.

It has been claimed that palladium NPs can be biosynthesized cost-effectively utilizing Saudi propolis. With an IC_50_ of 104.79 µg/mL, palladium NPs successfully cured MCF-7 ductal cancer (Al-Fakeh et al., 2021). These palladium NPs have been modified to treat MCF7 breast cancer cells using PVP-functionalized palladium. PVP-palladium NPs dramatically reduced the viability of human breast cancer MCF7 cells at increasing doses. Caspase3/7 enzymatic activity was hypothesized to be the mechanism for the system's induction of death by causing damage to mitochondrial membrane potential and nuclear DNA (Ramalingam et al., 2020).

### 3.2. Non-noble metals-based nanoparticles for cancer therapeutics

Non-noble metals despite their prone nature to oxidation have many advantages like they are low cost, abundant, and possess good conductivity. The application of these metals in cancer therapeutics is described below.

#### 3.2.1. Magnetic nanoparticles (iron/nickel)

The manipulation of MNPs is possible due to the use of external magnetic fields. Magnetic materials such as iron, nickel, or cobalt and functional chemicals are the most common components of these particles (Edis et al., 2021). A high-frequency magnetic field can elevate the temperature of the tumor to 40–46 °C by generating heat from these NPs. Another noteworthy potential of MNPs is their ability to combine heat (hyperthermia) with drug release in cancer treatment (Dwivedi et al., 2020). In a study, MNPs containing DOX–gelatin cores and Fe_3_O_4_–alginate shells were used to deliver targeted anticancer drugs. Doxorubicin was used as a model drug and implanted in the gelatin core to achieve excellent encapsulation efficiency. Controlled drug release was achieved by using an outer magnetic film, which could target the tumor tissue. These NPs were observed in the nucleus of MCF-7 breast cancer cells with an external magnetic field. Using an external magnetic field, the NPs efficiently targeted MCF-7 breast cancer cells, and after six hours of incubation, they appeared in the nuclei of those cells. MCF-7 cell viability declined to 52.3% after 12 hours of treatment, with relative fluorescence intensity of 98.4% (Huang et al., 2020). Anticancer drug telmisartan (TEL) was delivered to Fe_3_O_4_ magnetic nanoparticles (MNPs) via grafted chitosan, a naturally occurring hydrophilic and biodegradable polymer. Drug-loaded MNP-CS (MNP–CS–TEL) exhibited pH-responsive controlled release properties. The cytotoxicity of MNP–CS–TEL against PC-3 human prostate cancer cells was dose-dependent. The anticancer effects of the anticipated nano-formulation were found to be significant (Dhavale et al., 2021). The capacity to load large amounts of pharmaceuticals and control drug release are two advantages of adding MNPs into self-assembled hybrid NPs. In this context, NPs made of nickel ferrite (NFO) have been used to deliver anti-cancer drugs. Poly vinyl alcohol/stearic acid hybrid with NFO-containing PEG was used for zidovudine (AZT) distribution. AZT was intracellular delivery confirmed via NFO-reinforced hybrid NPs (Joshy et al., 2020). An emphasis is also placed on the green chemistry-based fabrication of MNPs. The green synthesis of nickel oxide NPs in Arabic gum was reported. To test the cytotoxicity of nickel oxide NPs, they used the MTT method on cancer U87MG cell lines. For U87MG cancer cells, the IC_50_ of this compound was 37.84 g mL (Sabouri et al., 2021).

#### 3.2.2. Zinc oxide nanoparticles

One of the most common metallic NPs in the world is zinc oxide. Zinc oxide NPs have received a lot of attention recently because of their ability to produce ROS when exposed to light. Particles of zinc oxide can be modified chemically to increase their photocatalytic efficiency as well as their ability to generate ROS by a variety of methods including doping with metals, polymer modification, and organic photosensitizing agents. The improved antibacterial and anticancer activity of modified zinc oxide NPs can be attributed to their increased ROS generation efficiency (Sivakumar et al., 2018). The potential anticancer activity of the CUR-loaded zinc oxide NPs was investigated using the MTT assay on the rhabdomyosarcoma RD cell line, while their cytotoxic effects were assessed using the resazurin assay on human embryonic kidney cells. The large aspect ratio of ZnO structures was considered a factor in the NPs' increased cytotoxicity (Perera et al., 2020). In another study, it was reported that egg albumin was used in the biosynthesis of zinc oxide NPs. The system showed anticancer efficacy on MCF-7 as measured by the MTT assay, with considerable cytotoxicity and correspondingly reduced cellular viability. The prepared NPs induced ROS, which increased the regulated transcription of mRNA levels of apoptotic genes such as p53, bcl-2, caspase-3, and caspase-9 while drastically downregulating the expression of anti-apoptotic gene Bcl-2, according to a gene expression research (RT-PCR) and western blot analysis. The findings suggested that the nano system specifically suppressed MCF-7 gene expression via ROS damage and cell death induced by cytotoxicity (Vijayakumar et al., 2020).

#### 3.2.3. Copper nanoparticles

Copper is a necessary component of plant and animal metabolism. Naturally, it is a soft, moldable, and easy to bend substance, with high thermal and electrical conductivities. When compared to analogous other metals, such as platinum, silver, and gold, copper NPs are cheaper among the transition metals under consideration (Rayapa Reddy, 2017). Green synthesized is also emphasized for copper NPs in recent trends. Broccoli green extract was described as a green and environmentally friendly precursor for copper NP one-pot biosynthesis. The developed formulation was proven to be beneficial in the treatment of prostate cancer (Prasad et al., 2016). The cytotoxicity of a chitin-based silver and copper nanocomposite against human breast cancer (MCF-7) cells was investigated. The inhibitory concentration (IC_50_) of the system was found to be 31 mg. Further findings revealed an increase in ROS production, decreased antioxidant enzyme activity, and membrane integrity degradation, confirming the cellular cytotoxic effect of the copper–silver NPs-based nanocomposite (Solairaj et al., 2017).

#### 3.2.4. Cerium Oxide nanoparticles

Cerium oxide NPs surrounded by an oxygen lattice, have shown potential in a variety of applications. These can induce apoptosis in the cancer cells (Gao et al., 2014). Through oxidative activation of the JNK apoptotic pathway, cerium oxide NPs make pancreatic cancer more sensitive to RT. Cerium oxide NPs cause cancer cells to produce more ROS. The oxidation of thioredoxin 1 (TRX1), which results in the activation of apoptosis signaling kinase 1, was demonstrated to be triggered by ROS (ASK1). Enhanced JNK activation was thought to be the outcome of increased TRX1 oxidation, based on the increase in ASK1 activation after co-treatment with Cerium oxide NPs followed by RT (Wason, 2018).

#### 3.2.5. Titanium nanoparticles

Natural forms of titanium dioxide include the inert minerals, anatase, brookite, and rutile, all of which occur in varying degrees of abundance. There are a lot of approaches to synthesize titanium dioxide NPs, including physical and chemical. These methods of nanomaterial synthesis have some shortcomings, including cost, low biocompatibility, and several secondary toxicities, as well as substantial environmental biosafety problems. As far as NP synthesis is concerned, biogenesis has been suggested. The biogenesis of titanium dioxide NPs uses a variety of organisms, including bacteria, algae, fungi, and plant materials. Biogenic titanium dioxide NPs have a unique size, shape, and biochemical functional corona that allows them to execute therapeutic effects at the molecular level, such as anticancer, antibacterial, antioxidant, larvicidal, and photocatalysis (Ikram et al., 2021). The use of titanium dioxide NPs in photothermal therapy for a melanoma cancer model was described by Muhammad et al. In the *in vivo* model, the average tumor size in the mice getting titanium dioxide-PEG NPs with laser excitation treatment decreased significantly compared to the mice receiving laser therapy alone (Behnam et al., 2018).

#### 3.2.6. Magnesium nanoparticles

Superparamagnetic magnesium ferrite-based NPs are used as radiosensitizers. Bio-nanocomposites and NPs systems have been proposed to combat cancer (Ansari Moghaddam et al., 2017; Mangalampalli et al., 2019; Kgosiemang et al., 2020).

## 4. *In vivo* application of metal nanoparticles

Several studies have been reported to check the anticancer effect of the metal NPs on tumor growth within animal models. Sriram et al. demonstrated the anticancer efficacy of biologically produced silver NPs *in vivo* using Dalton's lymphoma ascites. Silver NPs enhanced survival duration in the tumor mouse model by almost 50% as compared to tumor controls. Additionally, silver NPs reduced the volume of ascitic fluid in tumor-bearing animals by 65%, restoring normal body weight. The tumor volume was around 7.3 mL in control mice but was considerably reduced to 2.6 mL in the group treated for 15 days with silver NPs at a dosage of 500 nM (Sriram et al., 2010). In another study, selective radio-sensitization of brain tumors using gold NPs was checked on mice. The successful treatment of brain cancers such as glioblastoma multiforme (GBM) is constrained in large part by the cumulative dose of RT that may be safely administered and by the blood–brain barrier (BBB), which prevents the systemic anticancer medicines from reaching tumor tissue. Hence, the developed NPs exhibited that mice treated with gold NPs followed by RT had a longer median survival time (28 days on average) compared to mice treated with RT for only 14 days (*p*=.011). In general, mice treated with dual modalities maintained a higher level of normal activity and lost less weight than untreated or single-modality mice. The authors implied the successful extravasation of the gold NPs into the brain as a result of tumor-induced rupture of the BBB, where it radio-sensitized tumor cells to RT, resulting in enhanced tumor cell death and survival (Joh et al., 2013). Bis(2,4-pentanedionato)copper(II) encapsulated chitosan NPs were analyzed on mice model with control group having 1200 mm^3^ of tumor volume. The dose of 2000 μg/kg of body weight of both non-targeted and targeted NPs reduced tumor volume to 600 mm^3^ and 125 mm^3^, respectively (Bhanumathi et al., 2018) (Table 3).

**Table 3.** Comparison of tumor growth with metal nanoparticles and clinical drugs.

Type of nanoparticle	Animal model	Dose of the nanoparticle system	Effect of nanoparticle system on tumor growth	Control group (drug)	Dose of drug	Effect of drug on tumor growth	References
Doxorubicin loaded superparamagnetic iron oxide nanoparticles	Mice	Equivalent to 0.64 mg/kg doxorubicin	63% reduction	Doxorubicin	5 mg/kg	38% reduction	Yu et al. (2008)
RGD-modified apoferritin nanoparticles	Athymic nude mice	Equivalent to 10 mg/kg doxorubicin	89.6% tumor growth inhibition	Doxorubicin	10 mg/kg	74.0%	Zhen et al. (2013)
Platinum nanoparticles	K562-xenografted nude mice	Tumor volume reduction to ∼31.18 ± 3.42%	1 mg/kg	Cisplatin	1 mg/kg	Tumor volume reduction to 53.72 ± 5.60%	Zeng et al. (2020)
Folic acid- and berberine-loaded silver nanomaterial	Mice (control group tumor volume of 1287.27 ± 0.12 mm^3^)	Tumor volume of 101 mm^3^	5 mg/kg body weight	Berberine	5 mg/kg body weight	Tumor volume of 470 mm^3^	Bhanumathi et al. (2018)

## 5. Risk factors involved in the clinical application of metal nanoparticles

**Figure 8.**
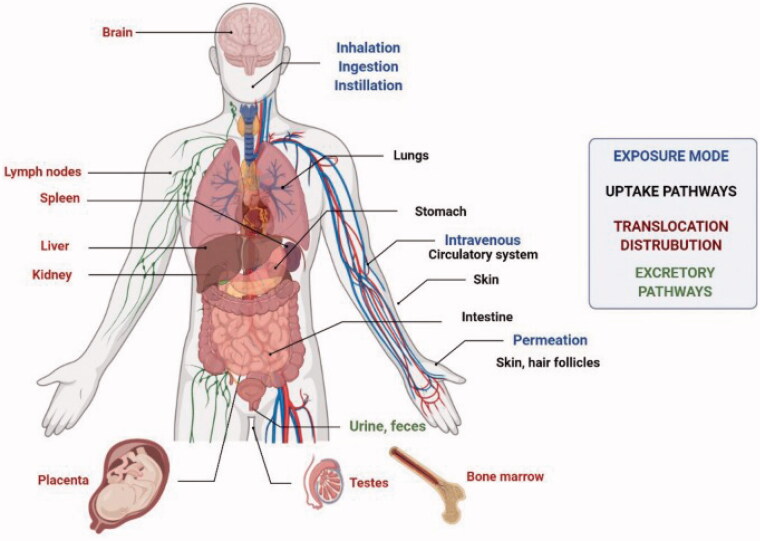
Exposure routes, ways of uptake, translocation, and distribution of NPs into the human body (Medici et al., 2021).

Nanoparticles have been found and produced in large numbers, but conventional criteria for limiting exposure and assessing their potential toxicity are lacking (Medici et al., 2021). Toxic effects are possible despite the particular advantages of nanomaterials due to their tiny size and high surface area which have been shown to boost reactivity with biological targets. Consumer and industrial products based on nanotechnology are nevertheless plagued by a slew of safety and sustainability issues (Grieger, 2019). Numerous problems regarding the safety and long-term sustainability of consumer and industrial goods based on nanotechnology remain unresolved (Najahi-Missaoui et al., 2021). Figure 8 depicts the various methods by which NPs might enter the body. Inflammation, genotoxicity, and organelle failure in cells are all directly linked to oxidative stress, which regulates the toxicity of NPs. The activation of oxidative enzymatic pathways results in the creation of nitrogen or oxygen-based free radicals, which in turn causes oxidative stress. Protein, DNA, and lipid damage, as well as mitochondrial and endoplasmic reticulum dysfunction and eventually apoptosis or ferroptosis, occur as a result of the relative imbalance or failure of the intracellular free radical scavenging mechanism's defense capability under prolonged oxidative stress (Reuter et al., 2010; Liao et al., 2020; Zhang et al., 2020).

The clinical application of the metal NPs can be achieved only if the risks and toxicity are controlled, from production to treatment. In a study, it was discussed that silver binds strongly to sulfur (both organic and inorganic) in natural systems (fresh and sea waters) as well as wastewater. Due to the decreased solubility of silver sulfide, sulfidation of silver NPs reduces their toxicity significantly, potentially limiting their short-term environmental impact (Liao et al., 2020). The manufacturing process of NPs is proposed by green synthesis utilizing plants, fungi, bacteria, and algae to avoid the high rate of harmful compounds and the severe environmental conditions used in chemical and physical processes. When compared to physical and chemical methods of synthesis, the green route of synthesis appeared to be safer and more environmentally friendly (Levard et al., 2012; Kalpana & Rajeswari, 2018). Copper NPs were synthesized by mixing copper acetate solution with *Eclipta prostrata* leaf extract without utilizing any surfactant or external energy. The antioxidant and cytotoxic properties of copper NPs made from *Eclipta prostrata* leaves extract were examined. *E. prostrata* leaf extract was reported as a good copper ion reducer, and the biosynthesized copper NPs were less harmful to the environment. The cytotoxicity of produced copper NPs against HepG2 cells was demonstrated in *in vitro* anticancer tests (Chung et al., 2017). The toxicity of NPs can also be reduced by using various polymers to coat metal NPs. To transfer cisplatin prodrug (DSP) to the bone, PEG coated NPs formed of a Zn^2+^ coordination polymer were coupled with a bone-seeking moiety, alendronate (ALN). *In vivo* biodistribution experiments revealed that DSP-Zn@PEG-ALN NPs intravenously delivered roughly fourfold more platinum to bone metastatic lesions than to healthy bones (He et al., 2017).

## 6. Conclusions and future perspective

Chemotherapy for cancer is still the most challenging area for academics looking for better clinical results. The use of targeted drug delivery for the treatment of cancer has the potential to improve efficacy while lowering the adverse and side effects. Nanotechnology has revolutionized medical research. The metal NPs are among the candidates for next-generation anticancer treatment. Metal NPs have been extensively studied due to their remarkable flexible physical and chemical properties. The current research has led to achieving various advantages with metal NPs including low cost, ease of synthesis, and the flexibility to control the form and size of the NPs. Green synthesis has also eliminated the risk of harsh and environmentally unfriendly compounds and solvents. Furthermore, due to their unique plasmonic properties, noble metal NPs provide a reliable means of tracking nano-complex therapeutic carriers within the body, allowing for a more efficient therapy with a lower chance of side effects than traditional therapies. These NPs would allow practitioners to diagnose and track the progress of treatment during treatment. Additionally, non-noble metal NPs are cost-effective and possess specific properties like hyperthermia and magnetic properties.

Several studies have demonstrated the efficacy of metal NPs as a future of cancer treatment, and many metal NP compositions are currently in preclinical and clinical trials. But a lot of concerns need to be addressed yet. The tumor imaging will be done with metal NPs to determine its exact stage, and tumor therapeutic methods will be developed in which the toxicity levels (prevalent in existing approaches) are completely eradicated. However, various criteria related to their manufacturing and use must be examined before they can be used in clinical trials. These precautions include control of techniques of preparation, repeatability, stability, dose, level of accumulation at the target and off-target sites, and, most critically, toxicological hazards. The regulatory agencies must play an important role in creating new criteria for clinical use of metal NPs in cancer treatment and drug delivery, as well as new approaches to evaluate the efficiency and safety measures of such NPs. On top of that, research scientists are struggling to come up with optimum amounts of phytochemical conjugated metal NPs for cancer patients and the most effective means of administering these doses. Metal NPs, on the other hand, will undoubtedly become a major clinical tool in the fight against cancer once these concerns are resolved.
